# The effects of physical games on memory and attention in children aged 5–6 years: a preliminary study

**DOI:** 10.3389/fped.2026.1860806

**Published:** 2026-10-27

**Authors:** Huihui Zhang, Yucheng Liu, Yifan Zuo, Jiabao Cui

**Affiliations:** 1 School of Sports, Shenzhen University, Shenzhen, Guangdong, China; 2 Qianzhen School, Longhua District, Shenzhen, Guangdong, China; 3 Sports Culture Research Base, Shenzhen University, Shenzhen, Guangdong, China

**Keywords:** 5–6-year-old children, attention, cognitive load theory, memory, physical games

## Abstract

**Background:**

This pilot study with class-level random assignment examined preliminary evidence on the associations between a structured physical game intervention and memory and attention in children aged 5–6 years.

**Methods:**

A 12-week physical game program based on cognitive load theory was designed and implemented, consisting of three 60-minute training sessions per week. The program included various game activities designed to promote memory and attention development supplemented by smart interactive motion data collection devices. A pilot study with class-level random assignment was conducted among 59 children aged 5–6 years. Two intact kindergarten classes were included, with one class assigned to the experimental group (*n* = 30) and the other to the control group (*n* = 29). The experimental group received the structured physical play intervention, whereas the control group participated in routine kindergarten physical activities. The class, rather than the individual child, was the unit of randomization. Before and after the intervention, children's memory and attention levels were assessed using the Wechsler Memory Scale (measuring picture memory, visual reproduction, visual recognition, digit span forward, and digit span backward) and the Schulte Grid test.

**Results:**

A 2 × 2 repeated-measures ANOVA showed significant Group×Time interactions for total short-term memory (*F*(1,57) = 14.33, *p* < .001, *η*p^2^ = .20), total immediate memory (*F*(1,57) = 7.84, *p* = .007, *η*p^2^ = .12), picture memory (*F*(1,57) = 6.10, *p* = .017, *η*p^2^ = .10), visual reproduction (*F*(1,57) = 18.40, *p* < .001, *η*p^2^ = .24), and digit span forward (*F*(1,57) = 5.26, *p* = .026, *η*p^2^ = .08). A significant Group×Time interaction was also observed for attention performance (*F*(1,57) = 8.17, *p* = .006, *η*p^2^ = .13). These findings indicated greater pre–post changes in the experimental condition than in the control condition for the outcomes examined.

**Conclusion:**

These findings provide preliminary evidence that a structured physical game intervention was associated with improvements in memory-related task performance and task-related attentional processing in children aged 5–6 years.

## 1 Background

Early childhood, particularly the preschool years between 5 and 6 years of age, is a critical “golden period” for cognitive neuroplasticity and the establishment of foundational behavioral patterns. During this period, the structure and functional connections of a child's brain are undergoing rapid maturation (1). Against this backdrop, attention and memory—as core components of cognitive development—play a crucial role in children's academic achievement, social adaptation, and lifelong learning abilities (2, 3). Attention is a multidimensional cognitive process that comprises various subcomponents, such as selective attention and visual search attention. Selective attention enables individuals to direct their cognitive resources selectively toward relevant information, while visual search attention supports individuals in actively detecting target stimuli in their environment. These subprocesses work in concert to facilitate children's focus on and processing of context-relevant information (4, 5). Concurrently, memory represents a complex cognitive system involving the temporary retention, recognition, and retrieval of information. Immediate memory refers to the immediate retention and reproduction of newly presented information, with minimal delay and no requirement for active manipulation. Short-term memory refers to the limited-capacity temporary maintenance of information over a brief interval. Working memory involves temporary maintenance together with active manipulation, reordering, or executive control. These constructs are related and partially overlapping rather than mutually exclusive; in the present study, they are distinguished primarily by task demands. Together, these cognitive processes support children's ability to acquire, process, and retain new information (6, 7). Research indicates that children aged 2–7 years are generally characterized by the preoperational stage of cognitive development, during which symbolic representation develops rapidly while logical operations remain limited (8). Therefore, supporting the development of attention and memory-related abilities in early childhood, and thereby promoting children's cognitive development—should be a key focus in the fields of developmental psychology and early childhood education (2, 7).

While extensive research has established the necessity of early cognitive intervention, current methodologies for enhancing attention and memory-related abilities in young children remain limited (9). Traditional training modalities often rely on sedentary, table-top tasks or rote memorization exercises; though targeted, these approaches are monotonous and unengaging, causing boredom or resistance in 5-to-6-year-olds and overlooking the developmental need for “play as a fundamental activity” in this age group (10). Therefore, there is a need to explore engaging and developmentally appropriate intervention strategies that integrate cognitive demands into children's natural play environments.

Physical games—defined as activities integrating movement, structured rules, and goal-directed challenges—offer a developmentally appropriate approach for promoting cognitive engagement in 5–6-year-old children. Unlike traditional physical activities that primarily emphasize metabolic demands, cognitively engaging physical activities require children to process information, remember rules or movement sequences, make decisions, and adapt responses during dynamic situations. Such cognitive demands may contribute to improvements in executive functions and related cognitive processes, including attention and memory regulation (2, 10, 11). From a physiological standpoint, research has been shown moderate exercise activates the prefrontal cortex and enhances executive functions; physical activity has been associated with increased secretion of brain-derived neurotrophic factor (BDNF), providing a plausible neurobiological basis for attentional control and memory consolidation (12–14). From a psychological perspective, physical games incorporate structured rules, goal-oriented tasks, and interactive elements that may facilitate children's active participation and provide cognitively stimulating learning experiences (15). During various game-based activities, children must focus on understanding rules, memorizing movement sequences, observing peer behavior, and responding rapidly to dynamic environmental shifts (16). A meta-analysis has explicitly distinguished between “cognitively engaging physical activities” (i.e., physical games) and “traditional aerobic exercises emphasizing metabolic demands” (e.g., running)； its findings indicate that the former yields superior improvements in executive functions, suggesting that cognitively demanding physical activities are significantly more effective at enhancing attentional control, inhibitory function, and memory than purely repetitive exercises (10). Consequently, physical games, as educational physical activities, play a significant role in the growth period of young children, both from physiological and psychological perspectives.

Recent advances in intelligent sensing technologies have also provided new opportunities for improving the implementation and evaluation of early childhood physical activities. Previous studies have demonstrated that wearable and sensor-based technologies can facilitate objective measurement of children's physical activity behaviors and provide valuable support for monitoring intervention processes (17, 18). Rather than replacing traditional physical play, technology-assisted systems can facilitate objective recording of children's behavioral responses, support intervention monitoring, and improve the accuracy and efficiency of data management in naturalistic educational settings (18). Accordingly, in the present study, the intelligent equipment was primarily designed as an auxiliary assessment and management system integrated into physical game activities, enabling systematic data collection, recording, storage, and management. Importantly, the cognitive effects investigated in this study are attributed to the structured physical game intervention itself, while the technological system served to support implementation and evaluation.

Accordingly, this study aims to empirically investigate the specific impacts of a 12-week physical game intervention on the development of memory and attention-related abilities in children aged 5–6 years. The findings provide preliminary evidence supporting the potential benefits of physical games for improving attention and memory performance in young children and may inform future intervention studies.

## 2 Materials and methods

### 2.1 Participants

Two intact kindergarten classes comprising 59 children aged 5–6 years were recruited, with the experimental group (*n* = 30) and the control group (*n* = 29). Informed consent was obtained from the legal guardians of all participating children. The research design and all associated assessment procedures were reviewed and approved by the Institutional Review Board (IRB) of Shenzhen University (Approval No. PN-202400029). The flowchart (Figure 1) is as follows:

**Figure 1:**
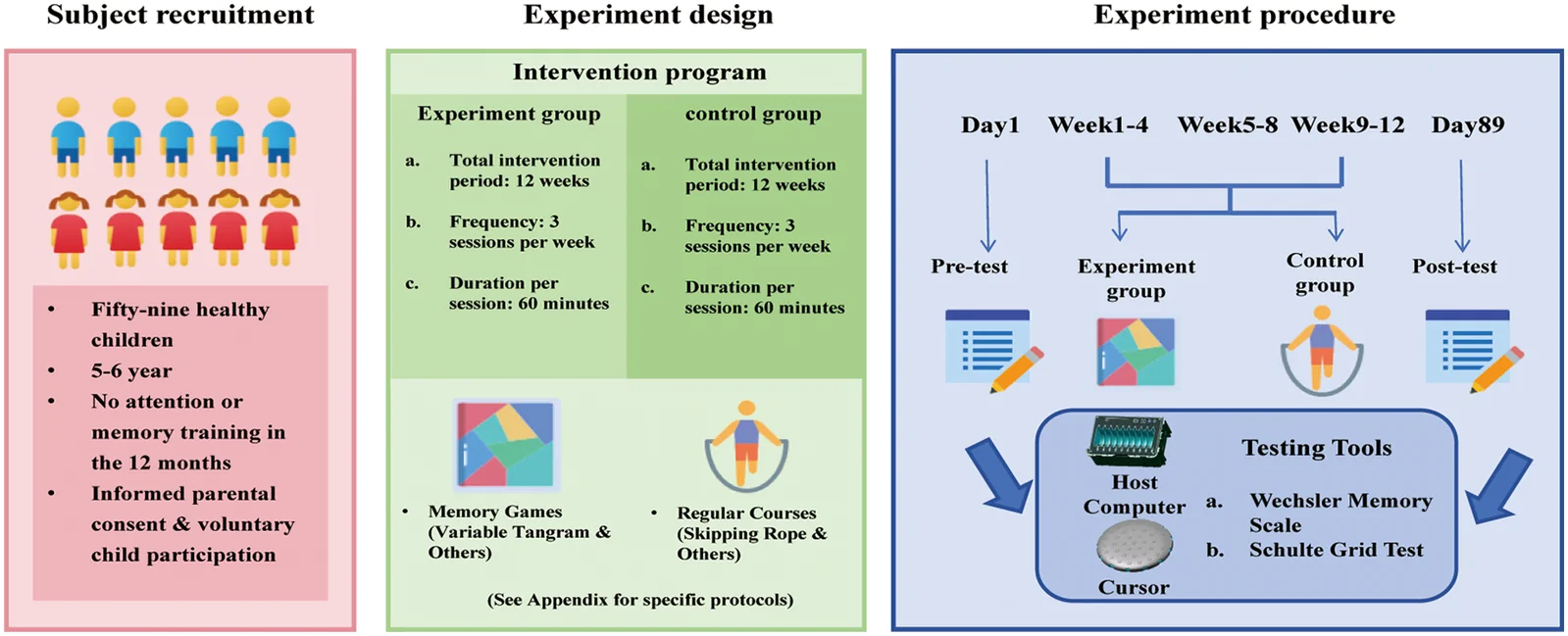
Experimental procedure. Reprinted with permission from Icons8 icon library by Icons8, licensed under the Icons8 Free License, https://icons8.com.

#### 2.1.1 Inclusion and exclusion criteria

The inclusion criteria for participants were as follows: (1) aged 5–6 years; (2) in good physical health, without reported developmental delays, cognitive impairments, or communication disorders according to kindergarten records, parent/teacher reports, and researcher observations, and able to participate in regular classroom activities; (3) no history of specialized memory or attention training or participation in extracurricular cognitive intervention programs within the past 12 months; and (4) voluntary participation with written informed consent by a parent or guardian. Participants were excluded based on the following criteria: (1) non-native nationality, due to potential confounding variables such as differences in language proficiency that affect comprehension; (2) presence of congenital diseases or medical conditions that renders the child unsuitable for extracurricular physical exercise; and (3) participation in external memory or attention-based training during the previous year. Furthermore, criteria for withdrawal or termination from the study included: (1) an attendance rate of less than 85%; (2) failure to cooperate with the instructor's curriculum arrangements during the intervention; (3) occurrence of adverse events preventing continued participation; (4) transferring to another school mid-study; and (5) failure to complete all data collection tasks or lack of engagement during assessment. Children who met the exclusion criteria were not separated from their classmates and continued to participate in regular kindergarten activities. Their data were excluded only from the present analysis.

#### 2.1.2 Research design and sample size

The study employed a pilot pre-test/post-test controlled design with class-level random assignment. The independent variable was a 12-week physical game intervention for children aged 5–6 years, while the dependent variables were memory and attention task performance. Sample size estimation was performed using G*Power software. Based on a two-tailed independent samples t-test with an assumed effect size of *d* = 0.67 derived from previous research (19), an alpha level of 0.05, and statistical power of 0.80, the estimated sample size was approximately 36 participants per group, which served as a reference for determining the overall study sample. The study selected two full-year senior kindergarten classes (Da-ban) from a kindergarten setting. After the baseline assessment was completed, an independent researcher who was not involved in the implementation of the intervention used a simple coin-toss procedure to assign the two intact classes to the experimental and control conditions, respectively. Thus, the randomization unit was the class, not individual children. The final sample comprised 59 children (30 in the experimental group and 29 in the control group), which was below this individual-level reference. This individual-level estimate served as a pragmatic planning reference and did not account for the clustering of children within classes or the small number of clusters; the study was therefore not powered for cluster-level inference. This limitation is discussed further in Section 4.4.

### 2.2 Intervention program

The intervention protocols for this study were developed based on cognitive load theory (CLT), which served as a theoretical framework guiding the design of age-appropriate physical games. Formulated by Sweller, CLT posits that total cognitive load consists of three components: intrinsic load, extraneous load, and germane load (20). The theory describes the relationship between limited working memory resources and long-term memory, suggesting that learning efficiency may be affected when task demands exceed available cognitive resources. In the context of this study, CLT was applied as a design principle rather than a directly tested mechanism. Specifically, intrinsic cognitive load was considered by adjusting task difficulty and physical demands according to children's developmental characteristics and prior experiences. Extraneous cognitive load was addressed through clear instructions, structured task organization, and simplified game procedures to reduce unnecessary processing demands. Germane cognitive load was conceptually considered through pedagogical elements, such as teacher guidance and peer cooperation, which assist children in better comprehending and engaging with the activities. Consequently, the physical games were designed to provide appropriate cognitive challenges and structured learning experiences. Cognitive load components were not directly measured in the present study, and the observed changes in memory and attention performance should be interpreted as the effects of a CLT-informed intervention design rather than evidence of changes in cognitive load itself.

Guided by the principles of CLT and incorporating various classical, early childhood physical games, a targeted intervention program was developed to enhance memory and attention. The program content underwent three rounds of Delphi expert consultations with 15 preschool education specialists (see Appendix for details). Based on expert feedback and input from frontline preschool teachers, the intervention was refined by augmenting game types, optimizing equipment and rules, and adjusting curriculum schedules. This iterative process yielded four distinct series of physical game interventions (Table 1), comprising 20 specific games: 10 memory-based games and 10 attention-based games (see Appendix for the detailed development process and game descriptions).

**Table 1:** Classification and description of the physical game intervention program.

Game Series	Individual Games	Primary Cognitive Domain
Flip and Match Series	Flip and Match (Color, Shape, Number); Running Flip and Match (Color, Shape, Number);	Memory
Whack-a-Mole Series	Individual Whack-a-Mole; Multiplayer Whack-a-Mole; Vertical Whack-a-Mole; Seated Whack-a-Mole	Attention
Finding Series	Picking Pearls; Treasure Hunt; Mini-Orienteering; Gift Delivery;	Attention
	Taking Veggies Home	Memory
Sticking Series	Find My Ball; Sticking Race;	Attention
	Find the Same; Sequential Sticking; Magic Tangram	Memory

The intervention dosage (three sessions per week, 60 min per session, for 12 weeks) was determined based on previous preschool physical activity intervention studies, which commonly employed structured interventions lasting approximately 12 weeks with 30–60 min of activity per session. Previous research has demonstrated the feasibility and developmental benefits of implementing structured physical activity programs within preschool settings (21, 22).

#### 2.2.1 Experimental equipment

Based on the functional design requirements of intelligent sports technology, our research team independently developed a suite of proprietary equipment. In this study, the intelligent sports equipment served as an auxiliary tool integrated into the physical game intervention, supporting activity delivery, real-time feedback, and objective recording of children's behavioral responses. The system consisted of two components: (1) interactive sensing equipment used during physical game activities and (2) a digital management platform used for data recording and intervention process management.

##### 2.2.1.1 Intelligent interactive sports information collection equipment

The interactive sensing equipment was designed to support the implementation of physical game tasks in kindergarten settings. During intervention sessions, children interacted with the sensing devices to complete game-related movements, while the system provided task-related feedback to support the execution of game procedures.

The primary functions of the sensory cursors include audiovisual feedback, real-time data collection, and asynchronous data transmission. In the present study, these functions were used to facilitate activity delivery and record intervention-related behavioral information. The primary advantage of this equipment is its ability to empower physical education through technology. By enhancing the interactivity and enjoyment of physical activities, it transforms the traditional exercise modalities for young children, bridging the gap between professional training and recreational play. Compared to conventional sports equipment, this system offers more comprehensive functionality and a richer user experience. Crucially, it provides a robust technical solution for the long-standing challenge of process evaluation regarding children's motor performance in naturalistic settings (Figures 2, 3).

**Figure 2:**
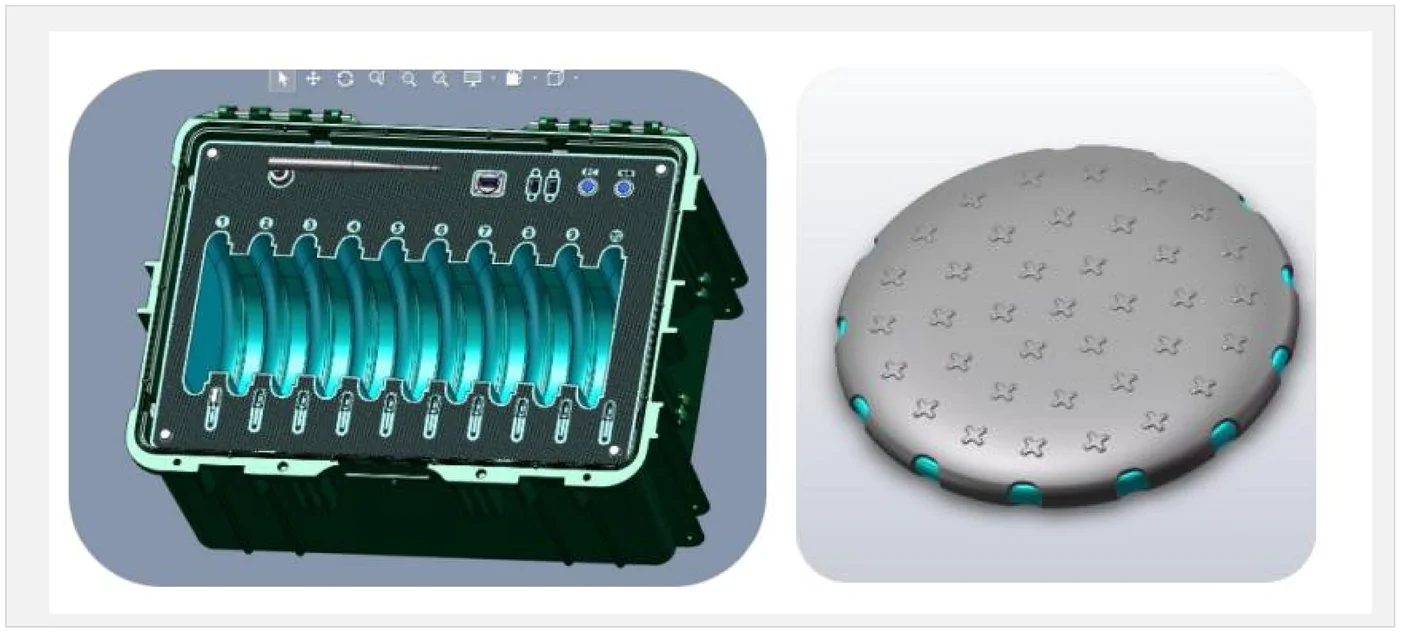
Device storage host and inductive cursor, and advantages of the inductive cursor system.

**Figure 3:**
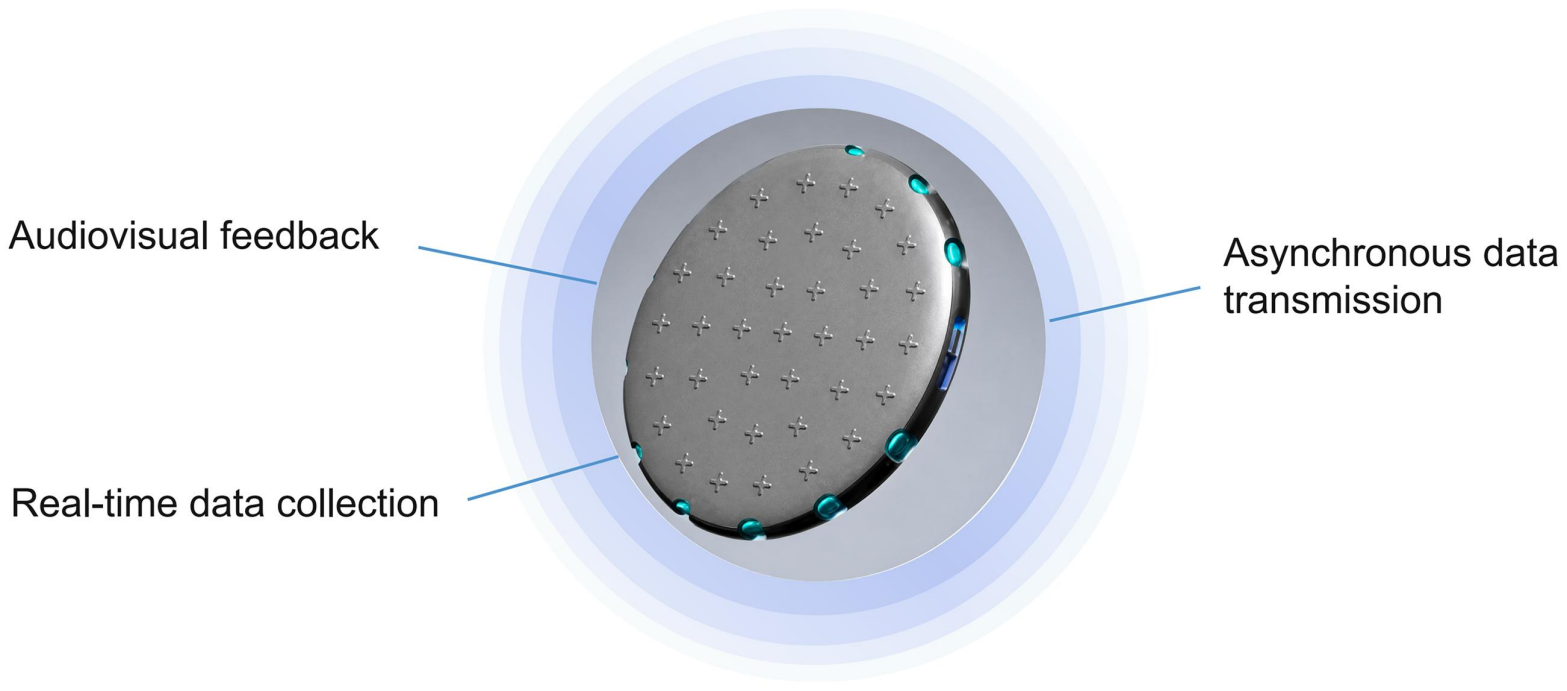
Functional advantages of the sensory cursor system.

##### 2.2.1.2 Integrated sports-education management platform

The digital management platform was developed to support intervention organization, data management, and monitoring of implementation fidelity.

During the intervention period, the platform was used to organize training sessions, record relevant activity information, and assist researchers in monitoring the consistency of intervention implementation across sessions.

The platform also facilitated the storage and management of collected behavioral data, providing technical support for subsequent analysis of intervention processes.

#### 2.2.2 Experimental group

The experimental group participated in three intervention sessions per week over a 12-week period. Each 60-minute session was structured into three distinct phases: a 20-minute preparatory phase, a 30-minute intervention phase, and a 10-minute concluding phase, ensuring adequate time for sports cognitive activities while preventing fatigue in young children.

Each session involved 30 children, who were divided into two subgroups of 15 children. The activities were conducted by a team of three qualified instructors: two intervention leads and one compliance and safety manager. Each intervention lead facilitated the physical games for a subgroup of 15 children. The compliance and safety manager monitored the overall activity environment, assisted with intervention implementation, and monitored participant safety throughout the sessions.

The intervention games were selected from four game series comprising 20 individual games. The selection and arrangement of games followed a progressive structure throughout the 12-week intervention period. During the adaptation phase (weeks 1–2), children were introduced to game rules and procedures, with 1–2 games conducted per session to facilitate familiarity. During the development phase (weeks 3–6), two games were implemented per session, with task requirements gradually adjusted to accommodate children's increasing familiarity with the game procedures. During the reinforcement phase (weeks 7–12), two games were conducted per session with gradually increased task complexity and intensity. The 20 individual games included in the four game series were rotated across sessions according to the intervention phase and children's adaptation level rather than following a fixed sequence throughout the intervention period.

To minimize potential within-session order effects (such as fatigue or reduced attention), the two subgroups performed the activities simultaneously using a crossover sequence. Specifically, the first subgroup engaged in the memory-based game intervention during the first half of the session and attention-based games in the second half, while the second subgroup followed the reverse chronological order.

#### 2.2.3 Control group

The control group followed the standard curriculum activities as prescribed by the kindergarten's pedagogical schedule, designed and implemented by the administration in accordance with national early childhood education guidelines. The control group followed its regular curriculum schedule with three 60-minute sessions per week over the 12-week intervention period. Activities were delivered by the class's regular physical education teacher, consistent with the kindergarten's standard staffing arrangement, reflecting the standard arrangement of the kindergarten rather than an additional study-specific modification. Thus, the experimental group and the control group were consistent in terms of the frequency and duration of physical activity; however, the content of the activities, teaching methods, and teacher assignments varied according to the respective intervention conditions.

The control group's physical education focused on traditional aerobic exercises and fundamental motor skill activities, such as running, organized campus walks, rope skipping, and basketball dribbling. Unlike the experimental group, these activities followed conventional teaching methods and did not include structured cognitive-motor challenges, technology-supported feedback, or interactive digital components. However, we acknowledge that basic motor activities may still involve elementary levels of visual-motor coordination, attentional focus, and rule-following, which are inherent to most structured physical activities.

### 2.3 Measurement instruments

#### 2.3.1 Memory assessment: wechsler memory scale (WMS)

The Wechsler Memory Scale-Revised for the Chinese population was developed as the Chinese revision of the Wechsler Memory Scale under the direction of Gong Yaoxian (23). The WMS-RC includes multiple memory-related tasks assessing different aspects of memory functioning.

In the present study, the selected WMS-RC subtests were used as behavioral measures of performance on specific memory-related tasks rather than as a standardized or clinical measure of general memory ability. Given the developmental characteristics and limited testing endurance of children aged 5–6 years, only selected subtests were administered instead of the complete battery.

Picture memory, visual reproduction, and visual recognition were used to assess the short-term retention and reproduction or recognition of visually presented information. Digit span forward was used to assess immediate verbal retention and reproduction, whereas digit span backward was used to assess a task requiring temporary retention and active reordering, which has a stronger working-memory component. These tasks were related but were not treated as interchangeable indicators of the same construct. These tasks have been used to assess specific memory-task performance in children and preschool-aged populations (24, 25).

All analyses were conducted using raw scores obtained from each subtest, with higher scores indicating better task performance. In the present study, Total Short-Term Memory was operationally defined as the sum of raw scores on Picture Memory, Visual Reproduction, and Visual Recognition. Total Immediate Memory was operationally defined as the sum of raw scores on Digit Span Forward and Digit Span Backward. These were the composite outcome definitions used in the analyses and should not be interpreted as standardized WMS-RC domain scores. To address the distinct demands of the two digit-span tasks, forward and backward digit span were also analyzed separately. Total Immediate Memory should therefore be interpreted as an operational digit-span composite rather than as a pure measure of immediate memory or working memory.

It should be noted that the selected WMS-RC subtests were originally developed within a broader memory assessment framework, and preschool-specific normative data are limited. Therefore, the scores were interpreted as indicators of memory-related task performance and changes following the intervention, rather than as standardized measures of general memory ability or clinical indicators.

#### 2.3.2 Attention assessment: schulte grid test

The computerized Schulte Grid test is a visual search-based assessment that records task completion time to evaluate visual attention and processing efficiency (26). As a classic methodology in cognitive psychology, visual search tasks require individuals to identify and locate specific targets within a complex environment, thereby providing a measure of the mechanisms underlying attentional allocation. Reaction time (RT) is one of the most established psychometric indicators in experimental psychology and can be used to reflect the efficiency of cognitive processing (27).

In this study, a computerized Schulte Grid task developed by the research team was used to obtain an objective measure of attention-related task performance. The task was implemented on the intelligent device system used in the present study. Before the formal assessment, standardized instructions were provided, followed by a practice trial to ensure that the children understood number recognition, ascending numerical sequencing, and the task procedures. During the formal assessment, children were required to identify and click the numbers from 1 to 25 in ascending order in a 5 × 5 grid. Task completion time was recorded automatically by the system and used as the primary outcome measure.

Performance on the Schulte Grid task requires the integration of several cognitive processes, including visual search, selective attention, numerical sequencing, processing speed, and response execution. Although numerical familiarity and sequencing ability may contribute to task performance, these abilities were not independently assessed in the present study. The standardized instructions and practice trial were therefore used to ensure that participants understood the task requirements. Accordingly, changes in completion time were interpreted as changes in task-specific visual search efficiency and attention-related processing performance, rather than as a direct measure of general attentional capacity.

### 2.4 Statistical analysis

Statistical processing and analysis of the raw data were performed using SPSS version 23.0. First, the Shapiro–Wilk test was employed to assess the normality of the data distribution prior to statistical inference; *p* > .05 indicated that there was insufficient evidence to reject the null hypothesis of normality, suggesting that the data were approximately consistent with a normal distribution.

To evaluate intervention effects, a 2 × 2 mixed-design repeated-measures analysis of variance (ANOVA) was conducted, with Group (experimental vs. control) as the between-subject factor and Time (pre-intervention vs. post-intervention) as the within-subject factor. The Group×Time interaction was considered the primary test of whether changes from pre- to post-intervention differed between the two groups. The main effects of Group and Time were also reported. Effect sizes were expressed as partial eta squared (*η*p^2^). Statistical significance was defined as *p* < .05. Data analysis and chart production were performed using GraphPad Prism 10.2.2 software (GraphPad Software, San Diego, CA, USA).

## 3 Results

### 3.1 Baseline characteristics of participants

A total of 68 children aged 5–6 years were initially recruited for this study. After excluding children who did not meet the intervention attendance criteria (experimental group, *n* = 4) and those who failed to meet the inclusion criteria (control group, *n* = 5; reasons included school transfer, diagnosis of autism spectrum disorder, and non-native nationality), the final sample size was 59 (experimental group, *n* = 30; control group, *n* = 29). Independent-samples t-tests and chi-square tests revealed no statistically significant differences between the two groups in gender distribution (*χ*^2^ = 0.013, *p* = .908) or mean age (t(57) = 0.035, *p* = .973). These findings indicate that no statistically significant baseline differences were detected between the two intact classes in the measured demographic characteristics (Table 2).

**Table 2:** Comparison of baseline characteristics between experimental and control groups.

Indicator	Experimental Group (*n* = 30)	Control Group (*n* = 29)	Statistic	*p*-value
Gender (Male/Female)	17/13	16/13	*χ*^2^ = 0.013	.908
Age (Years, *M* *±* *SD*)	5.78 ± 0.30	5.77 ± 0.35	*t*(57) = 0.035	.973

*M* *±* *SD* indicates Mean ± Standard Deviation; gender differences were analyzed using the chi-square test.

### 3.2 Effects of physical game intervention on memory performance

#### 3.2.1 Baseline memory characteristics

To examine baseline differences between the two groups prior to the intervention, independent-samples t-tests were conducted for memory indicators. Pre-test results (Table 3) showed no significant differences between experimental and control groups in any indicators of short-term memory (picture memory, visual reproduction, and visual recognition) or immediate memory (digit span forward and digit span backward) (*p* > .05). No significant baseline differences were detected between the two groups in the measured memory indicators.

**Table 3:** Comparison of baseline memory scores between experimental and control groups before intervention (*M* *±* *SD*).

Memory Types	Indicator	Experimental Group (*n* = 30)	Control Group (*n* = 29)	*t*-value	*p*-value
Short-Term Memory	Picture Memory	8.33 ± 2.29	8.41 ± 1.74	−0.151	.880
	Visual Reproduction	7.23 ± 2.22	7.34 ± 2.11	−0.197	.844
	Visual Recognition	8.80 ± 2.55	9.03 ± 2.83	−0.334	.739
	Total Short-term Memory Score	24.37 ± 4.00	24.79 ± 4.26	−0.396	.693
Immediate Memory	Digit Span Forward	7.73 ± 0.94	7.34 ± 1.08	1.474	.146
	Digit Span backward	2.83 ± 0.79	2.90 ± 0.62	−0.341	.734
	Total Immediate Memory Score	10.57 ± 1.17	10.24 ± 1.24	1.037	.304

*M* *±* *SD* indicates Mean ± Standard Deviation.

#### 3.2.2 Intervention effects on memory: group×time interaction

The effects of the 12-week intervention on memory performance were examined using a 2 × 2 mixed-design repeated-measures ANOVA, with Group (experimental vs. control) as the between-subject factor and Time (pre-intervention vs. post-intervention) as the within-subject factor. The primary outcome of interest was the Group×Time interaction, which directly tests whether changes over time differed significantly between the two groups. Total short-term memory and total immediate memory were treated as primary outcomes, whereas picture memory, visual reproduction, visual recognition, digit span forward, and digit span backward were treated as secondary outcomes (Table 4; Figure 4).

**Table 4:** Mixed-design repeated-measures ANOVA results for memory outcomes **.**

Outcome	Group *F*(1,57)	*p*	Time *F*(1,57)	*p*	Group×Time *F*(1,57)	*p*	Group×Time $\eta_{p}^{2}$
Primary outcomes							
Total Short-Term Memory	5.63	.021	154.90	<.001	14.33	<.001	.20
Total Immediate Memory	15.16	<.001	37.59	<.001	7.84	.007	.12
Secondary outcomes							
Picture Memory	4.94	.030	19.76	<.001	6.10	.017	.10
Visual Reproduction	8.02	.006	49.05	<.001	18.40	<.001	.24
Visual Recognition	0.03	.876	150.50	<.001	0.75	.390	.01
Digit Span Forward	15.96	<.001	29.14	<.001	5.26	.026	.08
Digit Span Backward	1.54	.220	10.44	.002	2.89	.094	.05

$\eta_{p}^{2}$ indicates partial eta squared. The Group×Time interaction was the primary test of differential change between the experimental and control groups.

**Figure 4:**
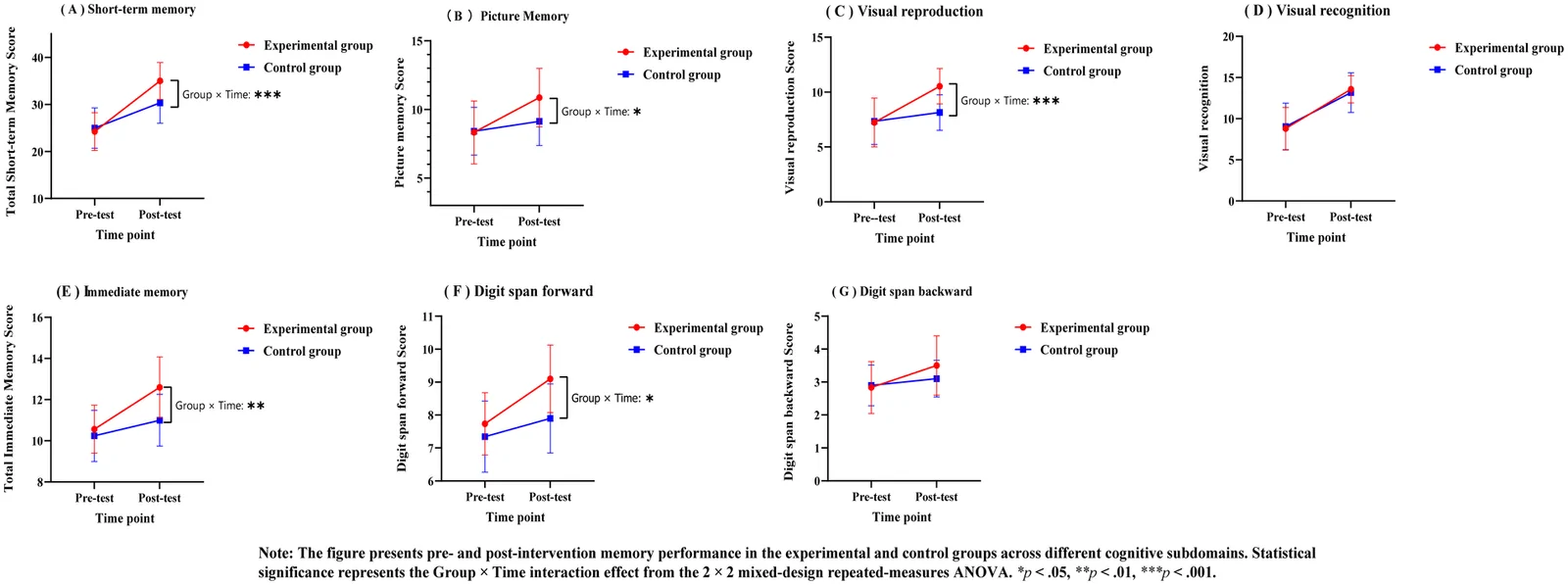
Effects of the physical game intervention on memory performance: results from a 2 × 2 mixed-design ANOVA.

For the primary outcomes, significant Group×Time interactions were observed for total short-term memory, *F*(1, 57) = 14.33, *p* < .001, $\eta_{p}^{2}$  = .20, and total immediate memory, *F*(1, 57) = 7.84, *p* = .007, $\eta_{p}^{2}$  = .12. These findings indicated that changes in both primary memory outcomes from pretest to posttest differed significantly between the experimental and control groups.

Among the secondary outcomes, significant Group×Time interactions were observed for picture memory, *F*(1, 57) = 6.10, *p* = .017, $\eta_{p}^{2}$  = .10; visual reproduction, *F*(1, 57) = 18.40, *p* < .001, $\eta_{p}^{2}$  = .24; and digit span forward, *F*(1, 57) = 5.26, *p* = .026, $\eta_{p}^{2}$  = .08. These results indicated that the changes in these memory components differed significantly between the two groups over the intervention period.

In contrast, no significant Group×Time interaction was observed for visual recognition, *F*(1, 57) = 0.75, *p* = .390, $\eta_{p}^{2}$  = .01, or digit span backward, *F*(1, 57) = 2.89, *p* = .094, $\eta_{p}^{2}$  = .05. Thus, the changes in these two memory measures did not differ significantly between the experimental and control groups.

Overall, significant differential changes were observed for both primary memory outcomes and three of the five secondary memory measures, whereas visual recognition and digit span backward did not show significant between-group differences in change over time. However, because only one intact class was assigned to each condition, these memory-related differences cannot be fully separated from class-level factors such as teacher and teaching style. They should therefore be interpreted as preliminary evidence of differential change rather than strong evidence of an intervention effect.

#### 3.2.3 Intra-group pre-post changes

Paired-samples t-tests were conducted to further examine pre–post changes in memory performance within each group. The experimental group demonstrated significant improvements across all seven memory measures (all *p* ≤ .003). In contrast, the control group showed significant improvements in visual recognition, total short-term memory, digit span forward, and total immediate memory (*p* ≤ .022), whereas no significant changes were observed in picture memory, visual reproduction, or digit span backward (*p* > .05). The pretest-to-posttest changes are illustrated in Figure 4.

### 3.3 Effects of the intervention on attention

#### 3.3.1 Baseline homogeneity test for attention

An independent-samples t-test revealed no significant difference between the experimental and control groups in pre-intervention Schulte Grid test scores (*t*(57) = 0.119, *p* = .906), indicating consistent baseline attention levels in both groups (Table 5).

**Table 5:** Comparison of baseline attention scores between experimental and control groups before intervention (M ± SD).

Indicator	Experimental group (*n* = 30)	Control group (*n* = 29)	*t*-value	*p*-value
Schulte Grid Test	222.57 ± 91.35	219.76 ± 90.04	0.119	.906

*M* *±* *SD* indicates Mean ± Standard Deviation.

#### 3.3.2 Intervention effects on attention: group×time interaction

The Group×Time interaction was used as the primary indicator of differential change between the two groups. A significant Group×Time interaction was observed for Schulte Grid task performance, *F*(1, 57) = 8.17, *p* = .006, $\eta_{p}^{2}$  = .13, indicating that the change in task performance from pre- to post-intervention differed significantly between the experimental and control groups. This finding suggests that the intervention condition showed better performance on the Schulte Grid task, reflected in more efficient visual search and selective attention-related processing. As discussed in the interpretation of the memory results, this result should be interpreted as preliminary rather than as evidence of a specific intervention effect (Table 6; Figure 5).

**Table 6:** Mixed-design repeated-measures ANOVA results for schulte grid task performance .

Outcome	Group *F*(1,57)	*p*	Time *F*(1,57)	*p*	Group×Time *F*(1,57)	*p*	Group×Time $\eta_{p}^{2}$
Schulte Grid Task	4.07	.048	18.59	<.001	8.17	.006	.13

$\eta_{p}^{2}$ indicates partial eta squared. The Group×Time interaction was the primary test of differential change between the experimental and control groups.

**Figure 5:**
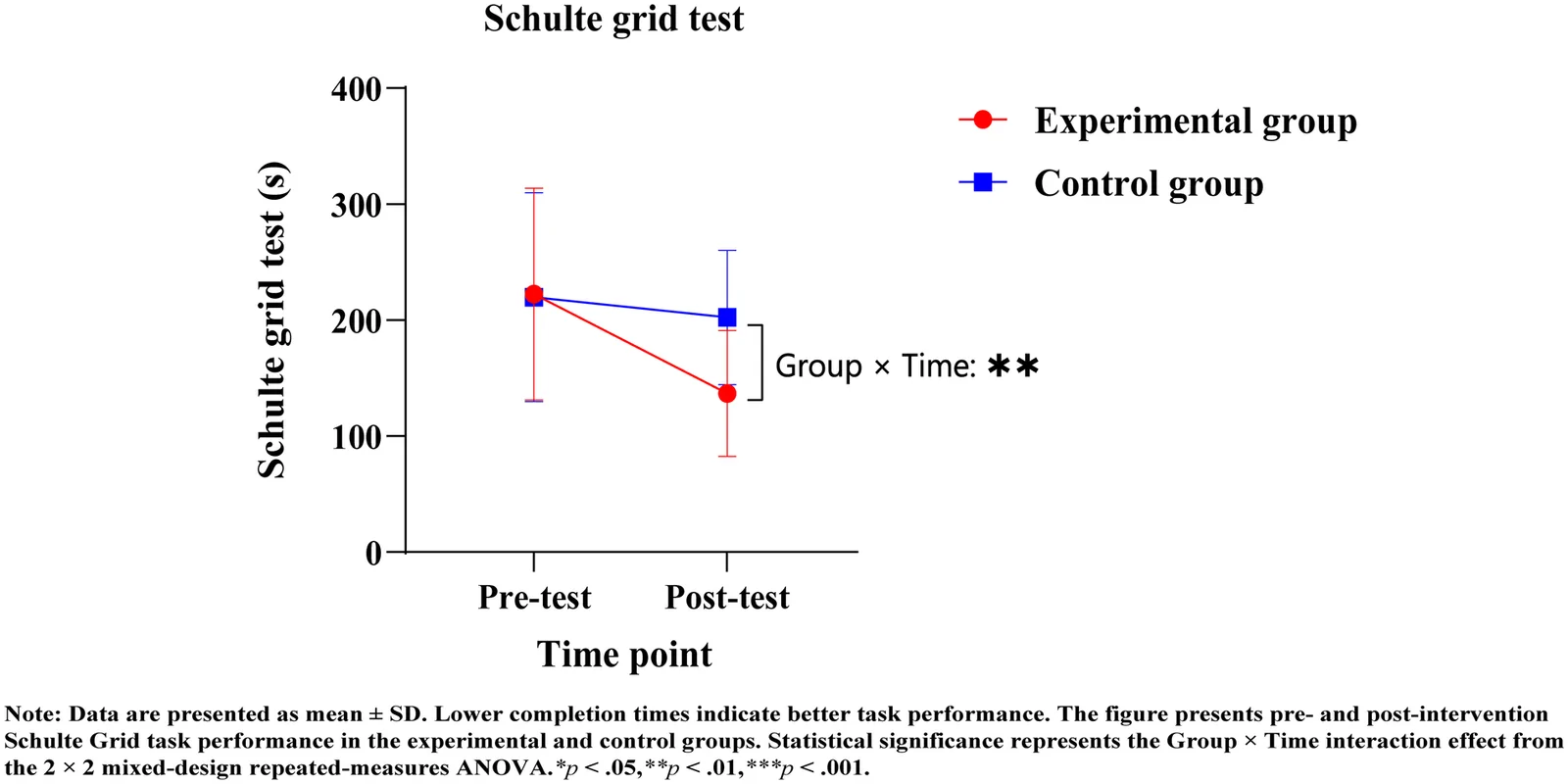
Changes in Schulte Grid task performance by group and time.

#### 3.3.3 Intra-group pre-post change

Paired-samples t-tests were conducted to further examine pre–post changes in Schulte Grid task performance within each group. The experimental group showed a significant reduction in task completion time from pretest (222.57 ± 91.35 s) to posttest (136.80 ± 54.28 s), *t*(29) = 5.368, *p* < .001, indicating improved attention-related task performance. In contrast, the control group showed a non-significant reduction in completion time from (219.76 ± 90.04 s) to (202.38 ± 57.75 s), *t* = 0.973, *p* = .339. The pretest-to-posttest changes are illustrated in Figure 5.

## 4 Discussion

The primary objective of this study was to examine changes in memory- and attention-related task performance following a 12-week structured physical game intervention informed by CLT among children aged 5–6 years. The experimental group showed greater pre–post changes than the control group in total short-term memory, total immediate memory, picture memory, visual reproduction, and digit span forward, as well as in attention performance. These findings provide preliminary evidence for the integration of cognitively engaging physical games into early childhood curricula to foster cognitive development.

### 4.1 Memory-related performance in the physical game intervention: targeted and differential changes

In this pilot sample, the intervention condition showed greater pre–post changes than the control condition across most memory indicators. These changes may be related to the intervention as implemented, including the CLT-informed game design, instructional procedures, and technology-supported feedback; they cannot be attributed to physical game content alone. Grounded in CLT, our game design adhered to the principles of “reducing extraneous load and optimizing germane load.” During the intervention, the use of intelligent equipment (sensory cursors and the management platform) provided immediate and clear audiovisual feedback, thereby minimizing children's confusion regarding rule comprehension. By embedding mnemonic tasks—such as remembering patterns, colors, numbers, or sequences—into contextualized physical activities like “memory match” or “gift delivery,” we directed the children's cognitive resources toward the germane cognitive load processes of encoding, storage, and retrieval. These activities may place demands on temporary information maintenance and, in some tasks, active manipulation or reordering. However, because working memory was not assessed as a separate composite outcome and the intervention components were not experimentally isolated, this interpretation should be considered tentative.

Notably, the intervention-related changes in this aspect of memory may have been less pronounced than those observed in other memory measures. Visual recognition involves the encoding, retention, and comparison of visual information, and repeated encoding may contribute to the precision of visual representations (28). However, the development of such representations is a gradual process that may be influenced by accumulated visual experience and the characteristics of the stimuli encountered (29). Thus, it is possible that the 12-week intervention was insufficient to produce a detectable between-group difference in visual recognition performance. This interpretation should be considered tentative, as the present study did not directly examine the mechanisms underlying changes in visual recognition.

In contrast, components of the intervention package involving active maintenance and mental manipulation may have been more closely aligned with specific memory processes. This interpretation is broadly consistent with findings from cognitive neuroscience suggesting that physical activity combined with cognitive challenges may influence neural plasticity and benefit memory performance (30). The changes observed in the intervention as implemented therefore raise the possibility of differential patterns across specific memory processes rather than uniform changes across all memory components. Future research should further examine these differential effects and investigate the cognitive and neural mechanisms that may contribute to them.

### 4.2 Attention-related performance in the physical game intervention

The intervention condition showed greater pre–post improvement in the Schulte Grid task than the control condition, reflected in more efficient visual search and task-related attentional processing within this specific testing context. This change may be related to the combined cognitive demands of the intervention package, which required children to engage in visual searching, target identification, and rapid decision-making. The intervention package, particularly the “seek and find” series, may have engaged similar processes; however, this interpretation is tentative because the intervention components were not experimentally separated.

Furthermore, the intelligent experimental equipment provided immediate audiovisual feedback (e.g., sound and light effects upon hitting a target), which may have enhanced task-related information processing and provided timely responses during physical game training. Consequently, the physical games in this study were not merely about “moving” but were characterized by “movement + cognition” (31). This approach shifted the children's attention from passive maintenance during static tasks to active regulation within complex, dynamic, and multimodal environments. This transition is crucial for developing executive control necessary for more advanced learning stages.

### 4.3 Intelligent technology empowerment and process evaluation

This study is the application of intelligent interactive equipment to address the long-standing challenge of “process evaluation” in early childhood physical education. Traditional assessment methodologies frequently rely on terminal outcomes—such as whether a child completes a race—while neglecting the subtle nuances of motor performance during the activity itself. Wearable technology using smart sensors can reliably measure young children's movements and physical activity levels in real time, providing an objective and continuous alternative to traditional observation-based assessments (32, 33). The intelligent equipment also supported intervention delivery and process management by providing task-related feedback and recording intervention-related activity information.

### 4.4 Limitations and future perspectives

First, the study used class-level random assignment and included only two intact kindergarten classes, with one class assigned to each condition. Although this approach was feasible within the participating kindergarten setting, the small number of classes means that class-level characteristics cannot be fully distinguished from the intervention condition. In addition, the sample size estimation was based on an individual-level reference and did not explicitly incorporate the clustering structure of class-level assignment. This consideration may limit the precision with which the findings can be generalized beyond the participating classes. The findings should therefore be regarded as preliminary. Future studies could address this issue by recruiting a larger number of classes across multiple kindergartens and incorporating the clustering structure into sample size planning and statistical analysis, such as through cluster-level or multilevel approaches.

Secondly, while we considered nationality as a factor in maintaining sample homogeneity, it is important to note that it should not be equated with a measure of language ability; future studies should directly assess language ability and include children from diverse linguistic and cultural backgrounds to enhance the generalizability of the findings. Moreover, regarding children with developmental delays or cognitive impairments, it is possible that they exhibit unique attention patterns and respond differently to physically active games that require cognitive engagement. Therefore, future research could consider cognitive studies of this population to explore the potential application of physically active game interventions in a broader population.

Furthermore, regarding the assessment tools: because the normative data and psychometric evidence for these WMS-RC subtests are relatively limited for the 5–6-year-old age group, the scores obtained in this study should be viewed as indicators of participants’ performance on specific memory-related tasks and changes following the intervention, rather than as standardized measures of general memory ability or indicators for clinical assessment. Future research should consider using assessment tools that have been specifically validated for preschool-aged children. A second assessment-related limitation concerns the Schulte Grid task. Performance on the Schulte Grid task may be influenced by familiarity with numbers and sequence-sorting ability, neither of which were independently assessed in this study. Although the study provided standardized operating instructions and practice trials, these task-specific requirements should still be taken into account when interpreting attention-related findings. Future studies could incorporate non-numerical measures of attention appropriate for this age group to reduce the impact of number and sequence-sorting requirements on the results.

Third, this study employed a 2 × 2 mixed-design repeated-measures analysis of variance to examine changes over time and differences between groups, with particular attention to the group×time interaction. However, because the same cognitive assessment measures were administered before and after the intervention, practice effects may not have been completely ruled out in either group. Future studies should, when feasible, use alternative assessment forms, incorporate additional assessment time points, or adopt other assessment approaches to better estimate and mitigate potential practice effects.

Finally some features of the intervention and assessment may have introduced additional sources of variation. Although intervention duration and frequency were matched between the experimental and control groups, they differed in instructor configuration, activity structure, cognitive demands, and the use of intelligent equipment. In particular, the audiovisual feedback and technological novelty of the equipment used only in the experimental group may have influenced children's engagement, motivation, or responsiveness independently of the physical game content. Therefore, the relative contributions of the physical game content, instructional conditions, technological features, and cognitively enriched task demands cannot be fully disentangled in the present design. Because these factors were not directly measured, their potential contributions to the observed cognitive outcomes cannot be determined. Future studies should consider more closely matched control conditions, standardized instructor arrangements, and direct assessments of children's engagement, motivation, and acceptability to better isolate the effects of the intervention content from those of instructional and technological features.

## 5 Conclusions

In summary, this pilot study with class-level random assignment examined the changes associated with a 12-week structured physical game intervention informed by CLT and supported by intelligent interactive technology. Compared with the control condition, the intervention condition showed greater pre–post changes in total short-term memory, total immediate memory, and attention-related task performance among children aged 5–6 years. These findings provide preliminary evidence that the intervention as implemented may support memory-related and attention-related task performance in early childhood.

These preliminary findings suggest the potential value of incorporating structured physical game activities, cognitive challenges, instructional procedures, and technology-supported feedback into early childhood physical activity programs. Future research involving multiple classes could further examine which intervention components contribute most to these outcomes and confirm the robustness and generalizability of the findings.

## Data Availability

The original contributions presented in the study are included in the article/Supplementary Material, further inquiries can be directed to the corresponding author.
