# Supplementary material for: The effects of physical games on memory and attention in children aged 5–6 years: a preliminary study
Source: Front Pediatr. 2026 Oct 27;14:1860806. doi: 10.3389/fped.2026.1860806 (PMC13748315; doi:10.3389/fped.2026.1860806)
Supplement: Supplementary file 1 [file Supplementaryfile1.docx]

**Appendix A:**

**Development Process of 12-Week Memory and Attention Sports Games**

1. **Design and Creation of Physical Games**

This study systematically organized research regarding the memory and attention of children aged 5–6 and synthesized existing relevant literature. Based on the Guidelines for Learning and Development of Children Aged 3–6 and the physiological and psychological developmental characteristics of attention and memory in this age group, 20 structured physical games were developed, consisting of 10 games each specifically targeting memory and attention. These games comply with the child development recommendations for kindergartens in China. In targeting children's attention and memory, the design skillfully integrates and innovates various game formats and incorporates intelligent sports equipment to ensure high-precision data collection. To guarantee the scientific rigor, relevance, and operational feasibility of the games, the final physical game intervention program was determined through three rounds of iterative validation by Delphi experts, supplemented by repeated trials that utilized the professional expertise and frontline teaching experience of practitioners in the field of preschool education.

2. **Expert Consultation and Validation Process**

This study employed the Delphi method, also known as the expert consultation method. It is a systematic predictive approach that relies on the collective knowledge and experience of a panel of experts to make informed judgments. Through a series of meticulously designed questionnaires combined with structured information feedback, this method aims to achieve a high degree of consensus among the expert group. As an evaluation tool that integrates both qualitative and quantitative analysis, the Delphi method plays a critical role in academic research and professional assessment. The specific steps are as follows:

1. Determination of the research theme.
2. Selection of experts.
3. Design of the expert questionnaire.
4. Execution of the first round of expert consultation.
5. Revision of the game program based on feedback.
6. Execution of the second round of expert consultation.
7. Further revision based on the secondary feedback.
8. Execution of the third round of expert consultation.
9. Finalization of the physical game intervention program.

**3 Expert Panel**

To ensure that the physical games designed in this study provide an effective intervention for the memory and attention of children aged 5–6, it was necessary to engage experts, scholars, and kindergarten directors with extensive experience in relevant fields to evaluate the proposed teaching protocols. The final expert panel comprised 10 individuals, including four Associate Professors or above, one Lecturer, one Assistant Professor, and four Kindergarten Directors. The educational background distribution—consisting of Doctoral, Master's, and Bachelor's degrees in a ratio of 5:2:3—met the rigorous requirements of the Delphi method. The professional profiles of the experts are summarized below (see Table 1):

**Table 1. List of Experts for Consultation and Validation**

| **Name** | **Affiliation** | **Degree** | **Title/Position** | **Research Direction** |
| --- | --- | --- | --- | --- |
| Chen** | Shenzhen University | PhD | Professor | Early Childhood Physical Education |
| Liu** | Shenzhen University;  SZU Nord Kindergarten | PhD | Associate Professor, Director | Early Childhood Health Education |
| Liu* | Northwest University | PhD | Associate Professor | Promotion of Early Childhood Physical Fitness and Health |
| Zhang* | Shenzhen University | PhD | Lecturer | Preschool Education |
| Cui** | Shenzhen Universityv | PhD | Associate Professor | Child and Adolescent Physical Education |
| Liu* | Chengdu University of Technology | Master | Lecturer | Early Childhood Physical Education |
| Liu** | Nanshan Kindergarten Affiliated to Shenzhen University | Bachelor | Director | Preschool Education |
| Chen* | Ziranli Education; International Childcare Center | Master | Director, Founder | Early Childhood Health Education |
| He** | Mingju Plaza Kindergarten | Bachelor | Director | Preschool Education |
| Yan* | Nanshan Kindergarten Affiliated to Shenzhen University | Bachelor | Deputy Director | Preschool Education |

**4 Statistical Results of the Delphi Expert Questionnaire**

**4.1 Expert Enthusiasm Coefficient**

Three rounds of expert consultation were conducted in this study. The recovery rates of the questionnaires for the three rounds were 67%, 100%, and 100%, respectively. With the exception of the first round, all rates reached or exceeded the 70% threshold, indicating a high level of enthusiasm among the experts. The rates of suggestions provided by the experts were 70%, 40%, and 20%, respectively, showing a progressive downward trend. This decline suggests a steady increase in the experts' consensus and approval regarding the designed physical games. Consequently, the finalized physical game intervention program demonstrates significant feasibility for enhancing the memory and attention of children aged 5–6 years (see Table 2).

**Table 2. Statistical Results of Expert Enthusiasm**

| **Round** | **Questionnaires Distributed** | **Questionnaires Recovered** | **Recovery Rate ( % )** | **Number of Experts Providing Suggestions** | **Suggestion Rate ( % )** |
| --- | --- | --- | --- | --- | --- |
| **Round 1** | 15 | 10 | 67 | 7 | 70 |
| **Round 2** | 10 | 10 | 100 | 4 | 40 |
| **Round 3** | 10 | 10 | 100 | 2 | 20 |

**4.2 Expert Authority Coefficient (Cr)**

To ensure the scientific rigor and integrity of this study, it is essential to evaluate the authority of the participating experts. A higher degree of expert authority correlates with greater reliability in the modifications proposed for the teaching quality evaluation indicators, thereby ensuring the scientific validity of the physical games designed to enhance children's memory and attention.

In this study, the level of expert authority within the field of early childhood physical education was measured using the "Expert Familiarity Scale (Cs)" and the "Judgment Basis Scale (Ca)." Specifically, the Expert Authority Coefficient (Cr) is calculated as the arithmetic mean of these two scales. The "Expert Familiarity" (Cs) reflects the experts' level of practical and theoretical knowledge in relevant fields, while the "Judgment Basis" (Ca) represents the standards and evidence upon which the experts base their judgments. During the three rounds of expert consultation, surveys were conducted to assess these dimensions, with results determined by the experts' self-evaluations (see Tables 3 and 4).

**Table 3. Expert Familiarity Scale (Cs)**

| **Level of Familiarity** | **Very Familiar** | **Quite Familiar** | **Moderately Familiar** | **Moderately Familiar** | **Very Unfamiliar** |
| --- | --- | --- | --- | --- | --- |
| **Cs Coefficient** | 0.9 | 0.7 | 0.5 | 0.3 | 0.1 |

**Table 4 Scoring Standards for Judgment Basis (Ca)**

| **Basis of Judgment** | **High** | **Medium** | **Low** |
| --- | --- | --- | --- |
| **Theoretical Analysis** | 0.3 | 0.2 | 0.1 |
| **Practical Experience** | 0.4 | 0.3 | 0.2 |
| **Knowledge of Peers** | 0.2 | 0.1 | 0.05 |
| **Expert Intuition** | 0.1 | 0.06 | 0.02 |

The following statistical results were obtained by collecting data and calculating the expert authority coefficient (Cs+Ca/2=Cr). As shown in Table 5, the authority of the 10 experts was comprehensively evaluated based on their scores across four dimensions of judgment basis—“theoretical analysis,” “practical experience,” “knowledge of peers,” and “expert intuition”—as well as the average familiarity coefficients regarding “input quality,” “process quality,” and “outcome quality.” The calculations revealed that the authority coefficients for all experts exceeded 0.8 in the third round. According to academic standards, when the expert authority coefficient (Cr) reaches or exceeds 0.7, the expert is considered to possess high authority in the corresponding field. This indicates that the selected panel possesses significant professional expertise in the domain of early childhood physical games. Consequently, the expert group selected for this study fully satisfies the research requirements and is capable of providing scientific and authoritative guidance for the development of physical games for children.

**Table 5. Statistical Results of the Expert Authority Coefficient (Cr)**

| **Round** | **Basis of Judgment** | **Judgment Coefficient** | $\sum\text{Ca}$ | **Indicator Dimension** | **Expert Familiarity** | $\bar{\text{Cs}}$ | **Cr** |
| --- | --- | --- | --- | --- | --- | --- | --- |
| **Round 1** | **Theoretical Analysis** | 0.264 | 0.832 | **Input Quality** | 0.722 | 0.716 | 0.774 |
|  | **Practical Experience** | 0.363 |  | **Process Quality** | 0.805 |  |  |
|  | **Knowledge of Peers** | 0.138 |  | **Outcome Quality** | 0.621 |  |  |
|  | **Expert Intuition** | 0.067 |  |  |  |  |  |
|  | **Theoretical Analysis** | 0.271 |  | **Input Quality** | 0.736 |  |  |
| **Round 2** | **Practical Experience** | 0.370 | 0.855 | **Process Quality** | 0.823 | 0.746 | 0.801 |
|  | **Knowledge of Peers** | 0.145 |  | **Outcome Quality** | 0.680 |  |  |
|  | **Expert Intuition** | 0.069 |  |  |  |  |  |
|  | **Theoretical Analysis** | 0.276 |  | **Input Quality** | 0.741 |  |  |
| **Round 3** | **Practical Experience** | 0.379 | 0.882 | **Process Quality** | 0.852 | 0.768 | 0.825 |
|  | **Knowledge of Peers** | 0.154 |  | **Outcome Quality** | 0.711 |  |  |
|  | **Expert Intuition** | 0.073 |  |  |  |  |  |

**4.3 Statistical Basis for Indicator Screening**

**(1) Mean (**$\bar{\text{X}}$**)**

In this study, the importance of an indicator item is directly proportional to the mean value of the scores assigned by the experts. A higher mean ($\bar{\text{X}}$) value indicates a greater convergence of expert opinions and more prominent importance of the indicator. During the screening process, only items with a mean score of 4.0 or higher were retained; those scoring below 4.0 were excluded and were not included in subsequent questionnaire revisions or the final evaluation indicator system.

(2) **Coefficient of Variation (CV)**

The Coefficient of Variation (CV) serves as a metric to measure the coordination of expert opinions; a lower CV indicates higher uniformity in the experts' scoring of a specific indicator. In this study, the threshold for the CV was set at 0.25. When the coefficient of variation was 0.25 or less (CV≤0.25), it signified high consistency in the experts' evaluation, and the indicator was retained. Conversely, a CV exceeding 0.25 indicated significant divergence in expert opinions, leading to the exclusion of the indicator from subsequent revised questionnaires and the final set of evaluation indicators.

(3) **Kendall’s Coefficient of Concordance (W)**

Kendall’s Coefficient of Concordance (W) was used to quantitatively assess the consistency of the expert panel's evaluations across the entire set of indicators. The coefficient ranges from 0 to 1, with higher values representing greater consensus among experts. In this study, we ensured that the results of the coordination tests were statistically significant. Indicators were considered coordinated and included in subsequent analyses only when the coordination coefficient was significant (p < 0.05).

(4) Full-score Ratio (Ki)

This study adopts the definition of the Full-score Ratio (Ki) as referenced in the Standardization Law of the People's Republic of China. It reflects the proportion of experts who considered a specific item to be "very important" or "important." A higher ratio indicates that the item holds greater perceived importance among the experts. Therefore, the full-score ratio serves not only as a valuable supplement to the mean value but also directly indicates the hierarchical importance of the items.

**Table 6. Cut-off Values and Calculation Formulas for Statistical Indicators**

| **Item** | **Indicator** | **Cut-off Value** | **Calculation Formula** |
| --- | --- | --- | --- |
| **Concentration Degree** | Mean（$\bar{\text{X}}$） | $\bar{\text{X}}$ > 4 | $\bar{\text{x}}$ = (X_1_+X_2_+⋯+X_n_) n |
| **Coordination Degree** | Coefficient of Variation（*CV*） | *CV* < 0.25 | *CV* = *S /* $\bar{\text{x}}$ |
| **Degree of Importance** | Kendall’s *W* | 0≤*W*≤1; Higher values indicate greater coordination | *W* = 15S/*K²（N³-N*） |
|  | Full-score Ratio（*Ki*） | 0≤*Ki*≤1，Higher values indicate higher importance | *Ki* = (*X_1_+X_2_+⋯+X_n_*)/n |

**4.4 Results and Revisions of the First Round of Expert Consultation**

This study selected 15 experts from relevant fields and conducted a statistical analysis of their authority and enthusiasm coefficients, which met the experimental requirements. A total of 15 questionnaires were distributed in the first round of expert consultation, resulting in active responses from 10 experts. Under conditions of anonymity and without mutual knowledge of each other’s participation, the experts analyzed and evaluated the physical games designed for young children. Based on the identified deficiencies in the game designs, the 10 experts provided corresponding suggestions for deletions and modifications.

In this study, the mean scores assigned by the experts to each game item served as the primary basis for indicator selection. A mean score ($\bar{\text{X}}$) below 8 indicated that the specific game had limited intervention efficacy in enhancing the memory and attention of young children. Consequently, such items were excluded and were not included in the subsequent physical game questionnaires or the finalized list of intervention projects. The specific expert opinions and suggestions are summarized in Table 7.

**Table 7. Statistical Results of the First Round of Expert Questionnaire (Full Score = 10)**

| **Game Name** | **Subjectivity** | **Scientific Rigor** | **Targeting** | **Feasibility** | **Mean, (**$\bar{\text{X}}$**)** | **Training Target** | **Expert Recommendations** |
| --- | --- | --- | --- | --- | --- | --- | --- |
| **A1转动的时钟(Rotating Clock0** | 8 | 7 | 5 | 6 | 6.50 | **Memory** | **Delete** |
| **A2送礼物(Gift Delivery)** | 8.5 | 9 | 8.5 | 9 | 8.75 | **Memory** | **Retain** |
| **A3翻翻乐(Flip and Match)** | 9 | 9.25 | 9.5 | 8.5 | 9.06 | **Memory** | **Retain** |
| **A4障碍跑(Obstacle Race)** | 7.5 | 7 | 5.5 | 7 | 6.75 | **Attention** | **Delete** |
| **A5立面打地鼠(Vertical Whack-a-Mole)** | 8.75 | 9 | 9 | 9.5 | 9.06 | **Attention** | **Retain** |
| **A6个人打地鼠(Individual Whack-a-Mole)** | 8.5 | 8.5 | 9.25 | 9.5 | 8.94 | **Attention** | **Retain** |
| **A7多人打地鼠(Multiplayer Whack-a-Mole)** | 9 | 8.75 | 9 | 8.75 | 8.88 | **Attention** | **Retain** |
| **A8坐姿打地鼠(Seated Whack-a-Mole)** | 8.5 | 8.25 | 8.5 | 8.5 | 8.44 | **Attention** | **Retain** |
| **Teacher Staffing** | 1 person |  |  |  |  |  | **Recommend Increase** |

In this study, the mean scores assigned by the experts to each game item served as the quantitative basis for indicator selection. A mean score ($\bar{\text{X}}$) below 8 indicated that the game provided limited intervention efficacy in enhancing the memory and attention of young children; consequently, such items were excluded and were not included in the subsequent physical game questionnaires or the finalized list of intervention projects.

Specifically, among the aforementioned physical games, the mean scores ($\bar{\text{X}}$) for A1 Rotating Clock and A4 Obstacle Race were 6.5 and 6.75, respectively. As both scores fell below the threshold of 8, these items were deleted. The remaining six items, all of which had mean scores ($\bar{\text{X}}$) exceeding 8, were retained. Furthermore, the experts raised several issues and provided modification suggestions and corresponding solutions regarding the overall and individual design of the physical games, as detailed below:

**Introduce a question ：**

**(1) Lack of Novelty**

There is a notable lack of novelty in the current program; specifically, there are too few games targeting memory, and the types of games designed for attention are overly homogenous, making it difficult to sustain children's interest over a 12-week intervention. Five experts noted that after excluding "A1 Rotating Clock" and "A4 Obstacle Race" due to their predicted insignificant intervention effects, only two items remained for memory intervention. Furthermore, the attention-based games are all variations of the "Whack-a-Mole" series. Overall, the quantity is insufficient and the content is repetitive, necessitating an increase in both the number and diversity of physical games.

(2) **Lack of Diversity and Multisensory Elements**

Four experts pointed out a lack of diversity, noting that the retained games rely solely on "Whack-a-Mole" and "Gift" elements. Given that children are in a stage of rapid brain development, the application of diverse elements can enhance cognitive exploration. The integration of rich, interconnected elements has a significant effect on increasing cerebral activity. Therefore, the design of **physical games** should incorporate a wider array of integrated elements to broaden children's cognitive horizons.

(3) **Insufficient Physical Activity Intensity**

Four experts expressed concerns regarding insufficient physical activity, noting that in "A5 Vertical Whack-a-Mole" and "A6 Individual Whack-a-Mole," only one child participates at a time, leading to excessive sedentary waiting periods. Additionally, "A3 Flip and Match" and "A8 Seated Whack-a-Mole" are performed in a seated position, resulting in minimal bodily movement. Collectively, the long waiting times and low exercise intensity may compromise the final intervention efficacy. Consequently, subsequent game designs must focus on reducing waiting intervals and increasing physical activity intensity.

**Advice of modification:**

(1) **New Additions: Increasing the Quantity and Physical Intensity of Physical Games**

It is recommended to increase the number of **physical games** based on the existing framework. Given the current scarcity of games targeting memory, additional items focusing on memory intervention should be incorporated. This can be achieved by adapting existing games, introducing novel gameplay mechanics, or enriching game elements with themes such as "food," "animals," "geometric shapes," and "colors." Such thematic integration allows children to immerse themselves in corresponding scenarios, thereby enhancing the richness and engagement of the games. Furthermore, to improve physical activity intensity, the games should incorporate longer running distances and diverse motor skills—such as "squatting," "transporting," and "pasting"—to increase the overall physical workload for the children.

(2) **New Additions: Increasing Teaching Staff to Optimize Intervention Efficiency**

The addition of a second intervention teacher is proposed. Due to the large number of participants in kindergarten settings, a single instructor often struggles to provide individual attention to every child while simultaneously managing the experimental intervention. By adding a second teacher to co-facilitate the tasks, the efficiency of the experimental intervention can be significantly improved. With two teachers delivering the same intervention content simultaneously, the children's waiting time can be reduced by half. This arrangement also enhances the supervision of children’s physical safety and ensures a higher standard of instructional quality.

**4.5 Results and Revisions of the Second Round of Expert Consultation**

Following an in-depth analysis of the feedback from the first round of expert questionnaires and in accordance with the established criteria, several **physical games** that did not meet the requirements were excluded. Concurrently, specific game contents were added, deleted, or modified based on expert feedback. These refinements led to the development of the second-round questionnaire. The revised second intervention protocol comprises a total of 18 **physical games** for young children, categorized into four thematic series: the "Whack-a-Mole" series (4 items), the "Flip and Match" series (6 items), the "Sticking" series (4 items), and the "Finding" series (4 items). Among these, 10 games target attention intervention, while 8 focus on memory intervention.

The specific classroom intervention protocol is as follows: A total of 30 children participated in the intervention and were divided into two groups. The sessions were co-facilitated by two teachers (interveners), each responsible for leading a group of 15 children. The **physical game** intervention was conducted three times per week. During each session, two out of the four game series were selected for implementation. The two groups proceeded simultaneously using a crossover design within the session: Group 1 received the memory intervention during the first half and the attention intervention during the second half; conversely, Group 2 received the attention intervention first, followed by the memory intervention (see Table 8).

**Table 8. Statistical Results of the Second Round of Expert Questionnaire**

| **Category** | **Game Name** | **Subjectivity** | **Scientific Rigor** | **Targeting** | **Feasibility** | **Mean,**$\bar{\text{X}}$ | **Training Target** | **Expert Recommendations** |
| --- | --- | --- | --- | --- | --- | --- | --- | --- |
| **打地**  **鼠系**  **列(Whack-a-Mole Series)** | **B1个人打地鼠(Individual)** | 8.7 | 8.5 | 9.0 | 8.4 | 8.65 | **Attention** | **Retain** |
|  | **B2多人打地鼠(Multiplayer)** | 8.5 | 8.6 | 8.5 | 9.0 | 8.65 | **Attention** | **Retain** |
|  | **B3立面打地鼠(Vertical)** | 8.5 | 9.0 | 9.1 | 8.5 | 8.78 | **Attention** | **Retain** |
|  | **B4坐姿打地鼠(Seated)** | 8.7 | 8.2 | 8.5 | 8.6 | 8.50 | **Attention** | **Retain** |
| **翻翻**  **乐系**  **列(Flip & Match Series)** | **B5翻翻乐**  **(Color)** | 8.9 | 9.0 | 8.7 | 8.5 | 8.78 | **Memory** | **Retain with Revisions** |
|  | **B6翻翻乐**  **（Shape）** | 8.9 | 9.0 | 8.7 | 8.5 | 8.78 | **Memory** | **Retain with Revisions** |
|  | **B7翻翻乐**  **（(Number)）** | 8.9 | 9.0 | 8.7 | 8.5 | 8.78 | **Memory** | **Retain with Revisions** |
|  | **B8跑翻乐（Running, Color）** | 8.9 | 9.2 | 8.7 | 8.5 | 8.83 | **Memory** | **Retain with Revisions** |
|  | **B9跑翻乐（Running, Shape）** | 8.9 | 9.2 | 8.7 | 8.5 | 8.83 | **Memory** | **Retain with Revisions** |
|  | **B10跑翻乐（Running, Number）** | 8.9 | 9.2 | 8.7 | 8.5 | 8.83 | **Memory** | **Retain with Revisions** |
| **贴一**  **贴系**  **列(Sticking Series)** | **B11寻找我的球(Find My Ball)** | 9.0 | 8.7 | 8.5 | 8.8 | 8.75 | **Attention** | **Modify** |
|  | **B12贴贴赛(Sticking Race)** | 9.1 | 8.9 | 9.1 | 9.1 | 9.05 | **Attention** | **Retain New Addition** |
|  | **B13找相同(Find the Same)** | 9.0 | 9.0 | 9.2 | 8.9 | 9.03 | **Memory** | **Retain New Addition** |
|  | **B14顺序贴图(Sequential Sticking)** | 8.4 | 8.6 | 8.9 | 8.8 | 8.61 | **Memory** | **Modify** |
| **找一**  **找系**  **列(Finding Series)** | **B15找宝贝(Treasure Hunt)** | 8.8 | 8.8 | 8.9 | 8.5 | 8.75 | **Attention** | **Retain New Addition** |
|  | **B16捡珍珠(Picking Pearls)** | 8.9 | 9 | 8.4 | 8.7 | 8.75 | **Attention** | **Retain New Addition** |
|  | **B17迷你定向(Mini-Orienteering)** | 8.7 | 8.9 | 9.1 | 9.0 | 8.93 | **Attention** | **Retain New Addition** |
|  | **B18送礼物(Gift Delivery)** | 9.0 | 9.0 | 8.5 | 9.0 | 8.88 | **Attention** | **Retain** |
| **配备教师(Staffing)** | | 2 Personds |  |  |  |  |  | **Recommended Increase** |

In the second round of expert consultation, 10 questionnaires were distributed and 10 were recovered, achieving a recovery rate of 100%. Four experts provided valuable qualitative suggestions during this round. Statistical analysis revealed that the mean scores ($\bar{\text{X}}$) for all physical game items exceeded 8.0. This indicates that the proposed games are perceived to have a significant intervention impact on the memory and attention of young children. Consequently, all items were retained in the protocol. The specific modifications are detailed as follows:

**Introduce a question ：**

(1) **Homogeneous Migration: Optimization of the "Flip and Match" Series**

The "Flip and Match" series (B5–B10) in the second protocol was adapted from "A3 Flip and Match" in the initial version. In this revised version, we categorized the designs into different colors, shapes, and numbers to train the memory retention of children aged 5–6 regarding these specific visual stimuli. Furthermore, the format for B8–B10 was transitioned from a stationary "Flip and Match" to a "Running Flip and Match" mode. This requires children to engage in running or even navigating obstacles during the game, thereby increasing physical activity volume and strengthening physical exercise. This modification necessitates greater time and effort to complete the tasks, which plays a positive role in enhancing the stability and persistence of the children’s memory.

(2) **Supplementing Diverse Types: Construction of New Series**

In the second round of expert consultation, two new series were introduced. The "Sticking" series, comprising B11 "Find My Ball," B12 "Sticking Race," B13 "Find the Same," and B14 "Sequential Sticking," consists of four physical games focused on memory, thereby addressing the previous shortage of memory-based interventions. Additionally, a "Finding" series was established, featuring gameplay centered on categorizing cursors of the same color or items of the same type. Building upon "A2 Gift Delivery" from the first version, it was refined into B18 "Gift Delivery," alongside three new additions: B15 "Treasure Hunt," B16 "Picking Pearls," and B17 "Mini-Orienteering." These seven new items all yielded mean scores ($\bar{x}$) exceeding 8.0 and were consequently retained. These designs are projected to have a positive impact on enhancing both the memory and attention of children aged 5–6 years.

**Advice of modification:**

(1) **Modification: Enhancing the Flexibility of Route Design**

Two experts pointed out that the fixed route design for **B11 "Find My Ball,"** which utilized printed posters, presented practical limitations. They noted that children would likely memorize the specific routes associated with each "ball" after only one or two sessions, rendering the material non-reusable for longitudinal intervention. Consequently, it was suggested to replace the printed posters with long ropes to delineate routes. This modification allows for flexible adjustments to the path configurations, ensuring the game's sustainability and repeated utility throughout the program.

(2) **Modification: Optimizing Cognitive Engagement**

One expert proposed refining the rules for **B14 "Sequential Sticking"** to allow children to autonomously select their sticking patterns and complete tasks using their individual mnemonic strategies. This shift toward learner autonomy is intended to foster the integrated development of both cognitive reasoning and memory retention in young children.

(3) **New Addition: Optimizing the Teaching Staff Structure**

Two kindergarten teachers recommended expanding the staffing for the **physical game** curriculum to a three-person team. This configuration consists of one physical education (PE) teacher, one lead teacher, and one assistant teacher. Such a collaborative structure is expected to better ensure the instructional continuity and physical safety of the children during the intervention.

**4.6 Results and Revisions of the Third Round of Expert Consultation**

Following the first two rounds of expert demonstration and revision, the experts' opinions on the selection of physical game items gradually converged. This process led to the preliminary construction of a physical game curriculum system specifically designed to enhance the memory and attention of children aged 5–6. Nevertheless, recognizing that certain aspects required further refinement, a third round of questionnaires was distributed. The objective was to solicit additional expert insights until a widely recognized curriculum system—characterized by concentrated scoring and unified opinions—was established. In this final round, 10 questionnaires were distributed and 10 were recovered, maintaining a 100% recovery rate. The mean scores ($\bar{\text{X}}$) for all physical game items ranged between 8.88 and 9.35, indicating a high level of consensus and statistical consistency among the experts.

As illustrated in Table 9, based on the three rounds of expert consultation and revisions, four series of physical games were finalized as the core content of this study. Specifically, 10 games were designated for memory enhancement and 10 for attention enhancement. Notably, 95% of the physical game designs achieved mean scores ($\bar{\text{X}}$) exceeding 9.0 across the dimensions of subjectivity, scientific rigor, targeting, and feasibility. This high level of expert endorsement suggests that, on a theoretical level, the curriculum design of this study has a positive impact on enhancing the memory and attention of children aged 5–6 years.

**Table 9. Statistical Results of the Third Round of Expert Questionnaire**

| **Category** | **Game Name** | **Subjectivity** | **Scientific Rigor** | **Targeting** | **Feasibility** | **Mean(**$\bar{\text{X}}$**)** | **Training Target** | **Expert Recommendations** |
| --- | --- | --- | --- | --- | --- | --- | --- | --- |
| **打地**  **鼠系**  **列(Whack-a-Mole Series)** | **C1个人打地鼠(Individual)** | 9.1 | 8.9 | 9.2 | 9.0 | 9.05 | **Attention** | **Retain** |
|  | **C2多人打地鼠(Multiplayer)** | 9.1 | 9.0 | 9.0 | 9.3 | 9.10 | **Attention** | **Retain** |
|  | **C3立面打地鼠(Vertical)** | 9.3 | 9.2 | 9.5 | 9.4 | 9.35 | **Attention** | **Retain** |
|  | **C4坐姿打地鼠(Seated)** | 9.0 | 8.8 | 8.7 | 9.0 | 8.88 | **Attention** | **Retain** |
| **翻翻**  **乐系列(Flip & Match Series)** | **C5翻翻乐**  **(Color)** | 9.0 | 9.2 | 9.1 | 9.2 | 9.13 | **Memory** | **Retain** |
|  | **C6翻翻乐**  **（Shape）** | 9.0 | 9.2 | 9.1 | 9.2 | 9.13 | **Memory** | **Retain** |
|  | **C7翻翻乐**  **（(Number)）** | 9.0 | 9.2 | 9.1 | 9.2 | 9.13 | **Memory** | **Retain** |
|  | **C8跑翻乐（Running, Color）** | 9.0 | 9.3 | 9.2 | 9.2 | 9.18 | **Memory** | **Retain** |
|  | **C9跑翻乐（Running, Shape）** | 9.0 | 9.3 | 9.2 | 9.2 | 9.18 | **Memory** | **Retain** |
|  | **C10跑翻乐（Running, Number）** | 9.0 | 9.3 | 9.2 | 9.2 | 9.18 | **Memory** | **Retain** |
| **贴一**  **贴系列(Sticking Series)** | **B11寻找我的球(Find My Ball)** | 9.3 | 9.2 | 9.3 | 9.1 | 9.23 | **Attention** | **Retain** |
|  | **B12贴贴赛(Sticking Race)** | 9.3 | 9.0 | 9.1 | 9.1 | 9.13 | **Attention** | **Retain** |
|  | **B13找相同(Find the Same)** | 9.2 | 9.1 | 9.2 | 8.9 | 9.10 | **Memory** | **Retain** |
|  | **C14顺序贴图(Sequential Sticking)** | 9.3 | 9.1 | 8.9 | 9.2 | 9.13 | **Memory** | **Retain** |
|  | **C15百变七巧板(Magic Tangram)** | 9.2 | 9.4 | 9.3 | 9.0 | 9.23 | **Memory** | **Retain New Addition** |
| **找一**  **找系列(Finding Series)** | **C16找宝贝(Magic Tangram)** | 9.0 | 9.3 | 9.1 | 8.8 | 9.05 | **Attention** | **Retain** |
|  | **C17捡珍珠(Picking Pearls)** | 9.4 | 9.2 | 9.3 | 8.9 | 9.20 | **Attention** | **Retain** |
|  | **C18迷你定向(Mini-Orienteering)** | 9.3 | 9.0 | 9.2 | 9.1 | 9.15 | **Attention** | **Retain** |
|  | **C19送礼物(Gift Delivery)** | 9.5 | 9.2 | 9.2 | 9.2 | 9.28 | **Attention** | **Retain** |
|  | **C20运菜回家(Taking Veggies Home)** | 9.3 | 9.1 | 9.3 | 9.1 | 9.20 | **Memory** | **Retain New Addition** |
| **配备教师(Staffing)** | | 3 Person |  |  |  |  |  | **Confirmed Increase** |

Finally, based on expert feedback and practical considerations, the final revisions were implemented to establish the **physical game** curriculum system aimed at enhancing the memory and attention of children aged 5–6 years. This system will serve as the framework for the instructional intervention experiment in this study. The specific modifications are as follows:

(1) **Optimization of Game Equipment and Rules**

Regarding the game **"C11 Find My Ball,"** both rules and equipment were refined based on expert suggestions. First, fixed routes were replaced with variable paths, enhancing the game’s feasibility and engagement. Second, additional procedural constraints were introduced—such as "duck walking," "ball handling," and "relays"—to increase the physical intensity and complexity of the game, thereby achieving the objectives of intensive training.

(2) **Addition of "C15 Magic Tangram" and "C20 Taking Veggies Home"**

To enhance the specificity of the **physical games**, particularly in strengthening children's memory, two new memory-based games were added. This adjustment balances the number of memory-targeting and attention-targeting games, facilitating subsequent lesson planning and instructional scheduling.

(3) **Curricular Adjustments: Staffing and Organizational Model**

The staffing for the **physical game** sessions was increased from two to three teachers, with corresponding adjustments made to the instructional design and methodology. While maintaining the original session duration, the addition of a third teacher enhances organizational discipline, safety management, and emergency response capabilities, while also allowing for greater curricular diversity and guaranteed physical workload. Ultimately, the curriculum adopts a hybrid model of **"Physical Games + Conventional Physical Exercise."** The 30 participating children are divided into two groups: during the first half of the session, the lead teacher conducts conventional physical exercises with one group, while the assistant teacher supports the PE teacher in facilitating the **physical games** with the other; the two groups then switch activities for the second half of the session.

(4) **Refinement of Game Rules and Logistics**

Upon finalizing the number of participants, session times, and the overall curriculum schedule for the experimental classes, variables such as game duration, number of sets, teaching aids, and lesson plans were calibrated to meet the specific needs and environmental contexts of the different classes.

**Table 10. Classification and Description of the Physical Game Intervention Program**

| **Game Series** | **Individual Games** | **Primary Cognitive Domain** |
| --- | --- | --- |
| Flip and Match Series | Flip and Match (Color, Shape, Number); Running Flip and Match (Color, Shape, Number); | Memory |
| Whack-a-Mole Series | Individual Whack-a-Mole; Multiplayer Whack-a-Mole; Vertical Whack-a-Mole; Seated Whack-a-Mole | Attention |
| Finding Series | Picking Pearls;Treasure Hunt; Mini-Orienteering;Gift Delivery; | Attention |
|  | Taking Veggies Home | Memory |
| Sticking Series | Find My Ball; Sticking Race; | Attention |
|  | Find the Same; Sequential Sticking; Magic Tangram | Memory |

**Table 11. Detailed Specifications of the Physical Game Intervention Protocol**

| **Type** | **Physical Game Items** | **Game Introduction** |
| --- | --- | --- |
| **Memory-based Physical Game Series** | **Flip and Match (Color、Shape、Number)** | **Experimental Setup：The children sit on the floor with five photoelectric balls arranged in an arc in front of them. Specific colors, numbers or shapes are placed atop each ball, which is then covered by a small bucket. The interval between the balls is approximately 30 cm.**  ****Procedures & Instruction**：The children first observe and memorize the corresponding numbers, colors, or shapes assigned to the photoelectric balls for 10 seconds. Upon the teacher’s verbal cue (e.g., "Seven"), the children must flip the correct bucket based on their memory to identify the matching content and tap the ball to extinguish the light.** |
|  | **Running Flip and Match(Color、Shape、Number)** | ****Experimental Setup**：Five photoelectric balls are arranged side-by-side at the starting line, each topped with a distinct number, color, or geometric shape. A whiteboard is positioned at the finish line 10 meters away, displaying corresponding contents that are programmed to light up randomly.**  ****Procedures & Instruction**：The children first observe and identify the contents on the photoelectric balls in front of them. When a specific ball lights up randomly, the children must recognize the corresponding content and sprint to the whiteboard at the finish line to locate and tap the matching item to extinguish it.** |
|  | **Find the Same** | ****Experimental Setup****：**Two photoelectric balls are positioned at either end of the course to serve as the start and finish line markers. A reference chart is displayed on a whiteboard, and corresponding stickers are placed at the starting line.**  ****Procedures & Instruction****：**Standing at the starting line, the children first observe and memorize the patterns and their respective positions on the reference chart. Each child then selects a sticker, sprints to the whiteboard, and identifies the matching pattern to apply the sticker. After completing the task, the child rapidly returns to the starting line to continue the mission. The complexity of the stickers progresses from simple to intricate designs.** |
|  | **Sequential Sticking** | ****Experimental Setup****：**Six to eight stickers are arranged in a specific sequence at the starting line. A corresponding base chart is displayed on a whiteboard 8 meters ahead, where a photoelectric ball is installed for task confirmation.** ****Procedures & Instruction****：**The children first observe and memorize the sequential order of the stickers. Subsequently, they must pick up the first sticker in the sequence, sprint to the whiteboard, locate the matching position to apply the sticker, and tap the photoelectric ball to confirm completion. The children then return to the starting line to retrieve the next sticker in order, repeating the process until all items are correctly placed. This activity can be conducted as either an individual timed trial or a group relay race.** |
|  | **Magic Tangram** | ****Experimental Setup****：**Two reference posters are affixed to a white magnetic board (sized 1.5m ×1m). Two sets of seven magnetic shapes (tangrams) are placed behind a line 5 meters away. Two photoelectric balls are positioned behind the line to serve as the start and finish points for recording the completion time.** ****Procedures & Instruction****：**Two children first observe and memorize the target patterns. Subsequently, they sprint to the 5-meter line to retrieve the required magnetic shapes and return to the whiteboard to construct a "house" according to the reference pattern. This process is repeated until the construction is complete. The two groups compete to determine who can finish the task in the shortest amount of time.** |
|  | **Taking Veggies Home** | ****Experimental Setup****：**Three baskets of different colors are positioned at one end of the course to represent "homes." In the center of the area, 9–12 photoelectric balls are dispersed, each marked with a vegetable pattern corresponding to one of the basket colors. Matching small baskets are placed at the starting line.** ****Procedures & Instruction****：**Each child holds a small basket corresponding to the color of their designated "home" and sprints to the central area to identify matching "vegetable balls." The children must tap the ball to extinguish the light and "pick it up" to place it in their basket. Only one item can be transported per trip. After returning to the starting line, the child continues the mission; the first to successfully transport all assigned vegetables wins.** |
| **Attention-based Physical Game Series** | **Individual Whack-a-Mole** | ****Experimental Setup****：**Several photoelectric balls are randomly scattered across the floor, with a distance of approximately 3–5 meters between each ball. The balls are programmed to illuminate in a random sequence.**  ****Procedures & Instruction****：**A single child stands in the center of the field. The child must observe the surrounding area for lighting cues; as soon as a specific photoelectric ball illuminates, the child must rapidly sprint to the ball and extinguish it by tapping it with a hand or stepping on it.** |
|  | **Multiplayer Whack-a-Mole** | ****Experimental Setup****：**Twelve photoelectric balls are randomly positioned across the floor, with a distance of approximately 3–5 meters between each ball.**  ****Procedures & Instruction****：**Three children await the teacher’s verbal command before sprinting into the field from three different directions. The photoelectric balls are programmed to illuminate in a random sequence. The children must monitor the field for lighting cues; as soon as a ball illuminates, they must rapidly sprint to it and extinguish it by stepping on it. The teacher records the number of lights extinguished by each child to determine the final score.** |
|  | **Vertical Whack-a-Mole** | ****Experimental Setup****：Ten photoelectric balls are arranged in a staggered vertical and horizontal layout, with some mounted on the wall and others positioned on the floor. The interval between individual balls is approximately 1–2 meters, while the distance between floor-mounted and wall-mounted balls is 2–3 meters. The balls are programmed to illuminate in a random sequence.  ****Procedures & Instruction****：The children must monitor both the wall and floor for lighting cues. As soon as a specific photoelectric ball illuminates, the children must rapidly sprint to the ball and extinguish it by tapping it with their hand. |
|  | **Seated Whack-a-Mole** | ****Experimental Setup****：Six photoelectric balls per group are arranged in a semi-circular arc. The distance between each ball is approximately 1 meter. The balls are programmed to illuminate in a random sequence.  ****Procedures & Instruction****：The children sit in the center of the arc. They must monitor the surrounding photoelectric balls for lighting cues and rapidly use their hands to tap and extinguish any ball that illuminates. |
|  | **Find My Ball** | ****Experimental Setup****：Five to eight photoelectric balls are arranged in a parallel line on the floor, with a lateral distance of approximately 1 meter between each ball. The starting line is positioned 5–8 meters opposite the balls.  ****Procedures & Instruction****：Standing behind the starting line, the children observe and search for the target object corresponding to their instructions. Once the target is identified, they sprint toward the correct object, retrieve it, and return to the starting line. The activity is conducted as a competition to determine who can complete the task in the shortest time. |
|  | **Sticking Race** | ****Experimental Setup****：Six photoelectric balls are positioned beneath a chart and programmed in a sequential order from 1 to 6. ****Procedures & Instruction****：Standing behind the starting line, the children first pick up the No. 1 icon and sprint to the chart to identify the corresponding missing section. They must then tap the photoelectric ball beneath it (the ball will illuminate only if the selection is correct). After completing the task, the children rapidly return to the starting line to retrieve the No. 2 icon and continue the sequence. The activity is conducted as a competition to determine who can finish all tasks in the shortest time. |
|  | **Treasure Hunt** | ****Experimental Setup****：Six photoelectric balls are scattered throughout various corners of the classroom. The starting point and the finish line are positioned at different locations.  ****Procedures & Instruction****：Using a simplified map provided to them, the children must first locate the starting point and extinguish the photoelectric ball. Subsequently, they are required to find and extinguish all photoelectric balls at waypoints 1 through 6 in the designated sequence to complete the exercise. |
|  | **Picking Pearls** | ****Experimental Setup****：Sixteen photoelectric balls are randomly dispersed throughout the area, consisting of four balls each in red, yellow, purple, and green. ****Procedures & Instruction****：Four children, each carrying a small basket in red, yellow, purple, or green, participate simultaneously. The children must monitor the area; as soon as they identify a "pearl" ball that matches the color of their basket, they must retrieve it and place it inside. |
|  | **Mini-Orienteering** | ****Experimental Setup****：Six to eight control points are established within the field. Each point is equipped with a photoelectric ball identified by a specific number or color, collectively forming a simplified course. A navigation map marked with a sequential series of waypoints is positioned at the starting line. ****Procedures & Instruction****：The children hold the navigation map and must locate the corresponding points in the prescribed sequential order to extinguish each photoelectric ball. Upon completing all waypoints in the correct sequence, the children proceed to the finish line. This activity can be conducted as an individual timed trial for evaluation. |
|  | **Gift Delivery** | ****Experimental Setup****：Twelve photoelectric balls are prepared for the activity. "Gifts" in three colors (red, blue, and green) are placed in baskets of corresponding colors at three distinct starting positions, each marked by a photoelectric ball of the same color. The remaining nine photoelectric balls are randomly dispersed throughout the center of the field. ****Procedures & Instruction****：Three children depart simultaneously from their respective starting points. They must sprint to the central area to locate the photoelectric ball that matches the color of the "gift" they are carrying and deposit it there. Only one gift may be delivered per trip. The child who first successfully delivers all assigned gifts to the correct color-coded locations wins. |

**Table 12. Course Content Schedule for the Experimental and Control Groups**

| **Time** | | **Experimental Group** | **Control Group** |
| --- | --- | --- | --- |
| Week 1 | Session 1 | ①Individual Whack-a-Mole | Rhythmic Exercise |
|  | Session 2 | ① Individual Whack-a-Mole | Basketball Activity |
|  | Session 3 | ① Find the Same | Cycling |
| Week 2 | Session 1 | ① Treasure Hunt | Rope Skipping |
|  | Session 2 | 1. Multiplayer Whack-a-Mole;② Taking Veggies Home | Circuit Training |
|  | Session 3 | ①Multiplayer Whack-a-Mole;② Find the Same | Rhythmic Exercise |
| week 3 | Session 1 | ① Seated Whack-a-Mole;② Sequential Sticking | Rope Skipping |
|  | Session 2 | ① Mini-Orienteering;②Flip and Match (Number) | Cycling |
|  | Session 3 | ①Flip and Match (Color);② Seated Whack-a-Mole; | Basketball Activity |
| Week 4 | Session 1 | ① Picking Pearls;② Flip and Match (Shape) | Walking Tour of Campus |
|  | Session 2 | ① Treasure Hunt;②Flip and Match (Number) | Rope Skipping |
|  | Session 3 | ① Picking Pearls;② Running Flip and Match (Color) | Cycling |
| Week 5 | Session 1 | ① Gift Delivery;② Find the Same | Circuit Training |
|  | Session 2 | ① Find the Same;② Treasure Hunt | Rhythmic Exercise |
|  | Session 3 | ① Taking Veggies Home;② Gift Delivery | Basketball Activity |
| Week 6 | Session 1 | ① Sticking Race;② Taking Veggies Home | Free Play |
|  | Session 2 | ① Find My Ball;② Running Flip and Match (Number) | Object‑Carrying Jump Game |
|  | Session 3 | ① Running Flip and Match (Shape);② Sticking Race | Cycling |
| Week 7 | Session 1 | ①Running Flip and Match (Color);②Multiplayer Whack-a-Mole | Walking Tour of Campus |
|  | Session 2 | ① Find the Same;② Treasure Hunt; | Bunny Hop Game |
|  | Session 3 | ① Multiplayer Whack-a-Mole;② Sequential Sticking; | Rope Skipping |
| Week 8 | Session 1 | ① Magic Tangram;② Find My Ball | Running Class |
|  | Session 2 | ① Sticking Race;② Sequential Sticking | Free Play |
|  | Session 3 | ① Magic Tangram;② Treasure Hunt | Cycling |
| Week 9 | Session 1 | ① Vertical Whack-a-Mole; ② Taking Veggies Home | Circuit Training |
|  | Session 2 | ① Flip and Match (Color);② Mini-Orienteering | "Jumping Goat" game |
|  | Session 3 | ① Vertical Whack-a-Mole;②Flip and Match (Shape) | Basketball Activity |
| Week 10 | Session 1 | ① Picking Pearls;②Flip and Match (Number) | Rhythmic Exercise |
|  | Session 2 | ① Treasure Hunt;Flip and Match (Color) | Object‑Carrying Jump Game |
|  | Session 3 | ① Picking Pearls;② Taking Veggies Home | Walking Tour of Campus |
| Week 11 | Session 1 | ① Gift Delivery;② Sequential Sticking | Free Play |
|  | Session 2 | ① Find My Ball;② Find the Same | Rope Skipping |
|  | Session 3 | ① Treasure Hunt;② Running Flip and Match (Color) | "Jumping Goat" game |
| Week 12 | Session 1 | ① Picking Pearls;② Magic Tangram | Cycling |
|  | Session 2 | ① Taking Veggies Home;② Seated Whack-a-Mole | Circuit Training |
|  | Session 3 | ① Mini-Orienteering;② Flip and Match (Shape) | Bunny Hop Game |
